# The Development and Study of Some Composite Membranes Based on Polyurethanes and Iron Oxide Nanoparticles †

**DOI:** 10.3390/membranes12111127

**Published:** 2022-11-10

**Authors:** Luiza Madalina Gradinaru, Stelian Vlad, Romeo Cristian Ciobanu

**Affiliations:** 1 “Petru Poni” Institute of Macromolecular Chemistry, Gr. Ghica Voda Alley, 41A, 700487 Iasi, Romania; 2 Electrical Engineering Faculty, “Gheorghe Asachi” Technical University of Iasi, Dimitrie Mangeron Bd., 67, 700050 Iasi, Romania; 3 SC All Green SRL, I. Bacalu Street, 5, 700029 Iasi, Romania

**Keywords:** polyurethane membranes, iron oxide nanoparticles, magnetic composites, structure–properties relationship

## Abstract

To improve the performance of composite membranes, their morphology can be tailored by precise control of the fabrication methods and processing conditions. To this end, the aim of this study was to develop novel high-performance composite membranes based on polyurethane matrix and magnetic nanoparticles with the desired morphology and stability, by selecting the proper method and fabrication systems. These well-prepared composite membranes were investigated from the point of view of their morphological, physico-chemical, mechanical, dielectric, and magnetic properties. In addition, their in vitro cytocompatibility was also verified by the MTT assay and their cell morphology. The results of this study can provide valuable information regarding the preparation of magnetic polyurethane-based composite membranes that could be used to design some suitable devices with tailored properties, in order to improve the image quality in magnetic resonance imaging investigations and to suppress local image artifacts and blurring.

## 1. Introduction

Developments in nanotechnology have been exploited to realize innovative techniques for the preparation of novel membranes with custom-tailored properties, which are used in a wide variety of applications in medicine [1], filtration performance [2,3], agriculture [4,5], food industries [6,7], etc. Notwithstanding all these advances, there is still a need and opportunities for improving membrane fabrication for a multitude of applications and also for employment in new tasks. Over time, significant effort has been focused on the synthesis of well-defined polymers, better control of the mechanism of pore formation, advanced techniques for surface functionalization, development of novel fabrication processes, lower costs, and less volume required for installations [8]. However, the core concept of membrane performance lies in its final morphology, and the selection of materials and fabrication methods significantly affect membrane morphology [9]. Thus, the development of membrane technology cannot be separated from its material development. Appropriate membrane materials highly contribute to the successful fabrication of a high-performance membrane. However, choosing the right materials is critical for avoiding artifacts and reducing sensitivity in biomedical and diagnostic applications [10].

Thus, polyurethanes (PU) are an important class of polymers that are used in various applications due to their availability, manufacturability, and a broad range of chemical, mechanical and biological properties [11,12,13,14]. These polymers consist of alternating hard and soft domains, which exhibit several desirable properties due to the microphase separation. Hard domains, the crystalline part of PU, are responsible for mechanical strength, while soft segments are rubbery and give the polymer elastic behavior and flexibility [15]. Over time, PUs have been used by numerous researchers to develop various membranes due to their outstanding flux capability, high salt-rejection properties, and elevated hydrophilicity [16].

Use of different inorganic compounds in the structure of polymeric membranes has been growing, to improve the properties of the final membranes. Various inorganic nanoparticles such as TiO_2_ [15,17,18], F-SiO_2_ [19], Ag [20,21], ZnO [22], Al_2_O_3_ [23], etc. have been introduced as fillers into the polyurethane matrix to improve the properties of the final composite membranes. In addition to these, magnetic nanoparticles, especially iron oxide nanoparticles (IONPs), have attracted much interest due to their special properties, which can be used in various areas including drug-delivery systems in cancer therapy [24,25], magnetic resonance imaging [13,14,26], electromagnetic screening devices [27,28], or other electrical systems [29,30,31]. In general, composite materials are composed of a micrometer-sized dispersed phase of inorganic and organic compounds, and, thus, their physical properties are controlled by the cumulative rule of each component. Moreover, the large surface-to-volume ratio of the nanoparticles is the key factor for the novel properties compared to those of the corresponding bulk material. The presence of finely dispersed inorganic nanoparticles in the polymeric matrix has been proven to be very useful in the improvement of membrane properties and performances for targeted applications [13,14,32]. In addition, these composite membranes can provide enhanced physical, mechanical, and thermal properties for various aggressive environments and also could be a way to stabilize the polymer membrane [33]. There are a variety of materials and material combinations that could be used in this regard, with a focus on nanosized oxide materials such as magnetite (Fe_3_O_4_), maghemite (γ-Fe_2_O_3_), and hematite (α-Fe_2_O_3_) [34,35]. These magnetic nanoparticles can be produced using a variety of techniques [36,37] and are distinguished by a magnetic moment along with good chemical stability and low toxicity.

Thus, in order to improve the performance of the composite membranes, their morphology can be tailored by the precise control of the fabrication methods and processing conditions. Even though significant work has been done in this field in the last century, understanding the relationships between the membrane morphology and the fabrication parameters is still very difficult. Therefore, in the first part of this work, we tried to select the appropriate methods and membrane fabrication systems to produce composite membranes with the desired morphology and stability and to select the best method to achieve these properties in an efficient way for future research. Then, in the second part, these well-prepared composite membranes were investigated from the point of view of their morphological, physico-chemical, mechanical, dielectric, and magnetic properties. In addition, their in vitro cytocompatibility was also verified by the MTT assay and their cell morphology. These magnetic polyurethane-based composite membranes could be used for the preparation of some suitable devices to improve the image quality in magnetic resonance imaging investigations. Thus, local image artifacts and blurring could be suppressed using this device. 

## 2. Materials and Methods

### 2.1. Materials

Poly(1,4-butylene adipate) diol end-capped (PBA) and Poly(tetrahydrofuran) (Terathane polyether glycol) (PTHF) with Mn 2000 g/mol, 1,4-butanediol (BD), and N, N-dimethylformamide (DMF) were purchased from Sigma-Aldrich (Steinheim, Germany). The 4,4′-diphenylmethane diisocyanate (MDI) was obtained from Fluka (Steinheim, Germany) and was freshly distilled prior to use. Iron (III) oxide nanopowder (Fe_2_O_3_) of <50 nm particle size (IONPs), ethylene glycol (anhydrous 99.8%), and methylene iodide (99%) were also purchased from Sigma-Aldrich. All other chemicals were used as received without further purification.

### 2.2. Synthesis of Polyurethanes

**Scheme 1:**
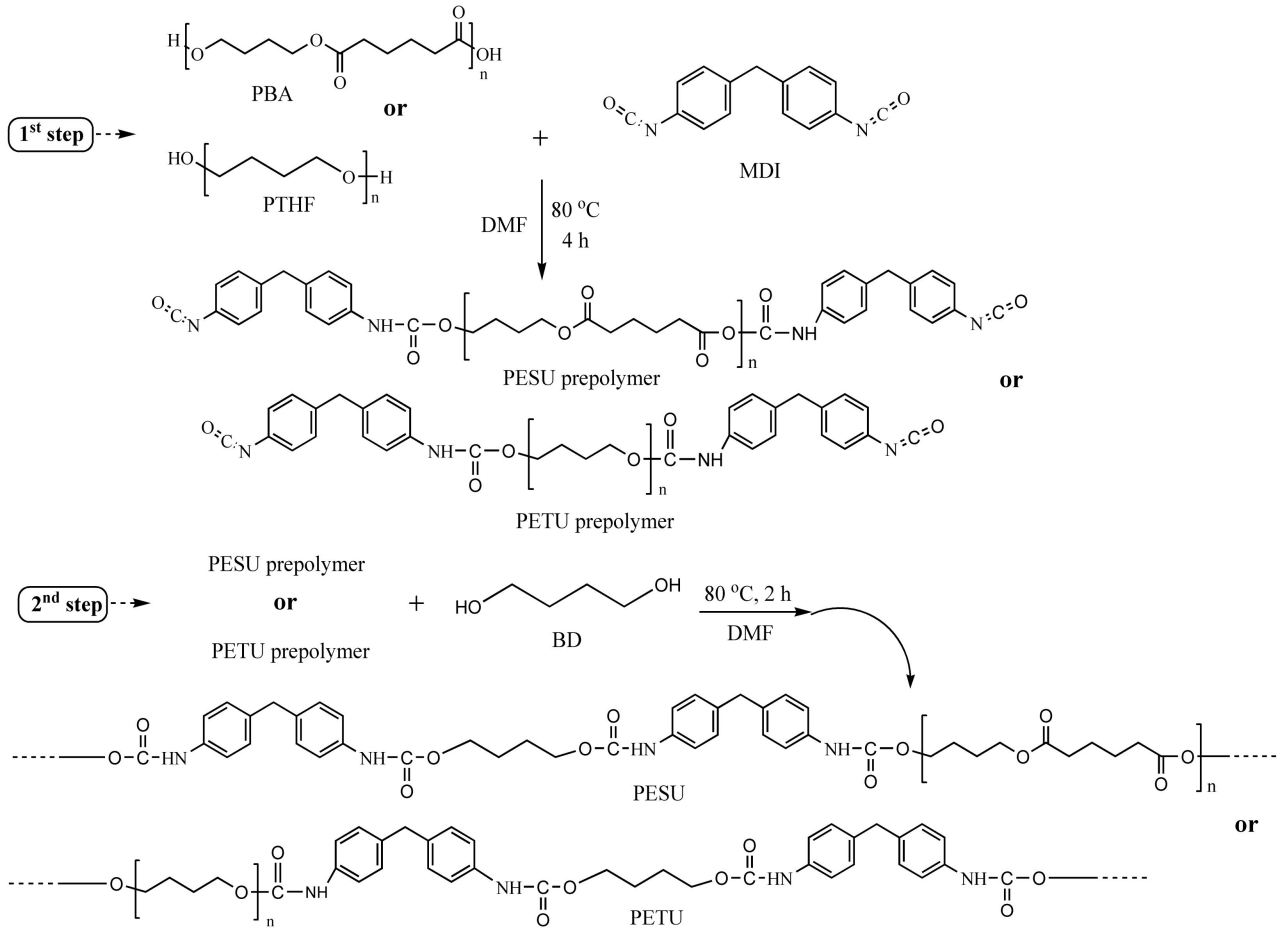
Synthesis pathway of polyester or polyether urethanes.

A polyester (PESU) and a polyether (PETU) urethane structures were selected for membranes’ formation and were synthesized using the classical prepolymer method in DMF as a solvent, a method that was described in our previous works [13,32]. At first, one equivalent of previously degassed PBA or PTHF and three equivalents of MDI were reacted at 80 °C for 4 h in a three-necked flask equipped with a mechanical stirrer, a heating oil bath, a dropping funnel, and an N_2_ inlet and outlet to prepare NCO-terminated polyurethane prepolymer. After two dilution steps, the prepolymer was reacted with two equivalents of BD as a chain extender at 80 °C for 2 h. The obtained viscous polyurethanes were then precipitated on a water bath and thoroughly washed with an excess of distilled water to eliminate the solvent and all water-soluble small-molecular-weight compounds. The precipitates were dried under vacuum for 24 h. The synthesis procedure of PESU or PETU urethanes is presented in detail in Scheme 1.

### 2.3. Preparation of Polyurethane-Based Membranes

**Scheme 2:**
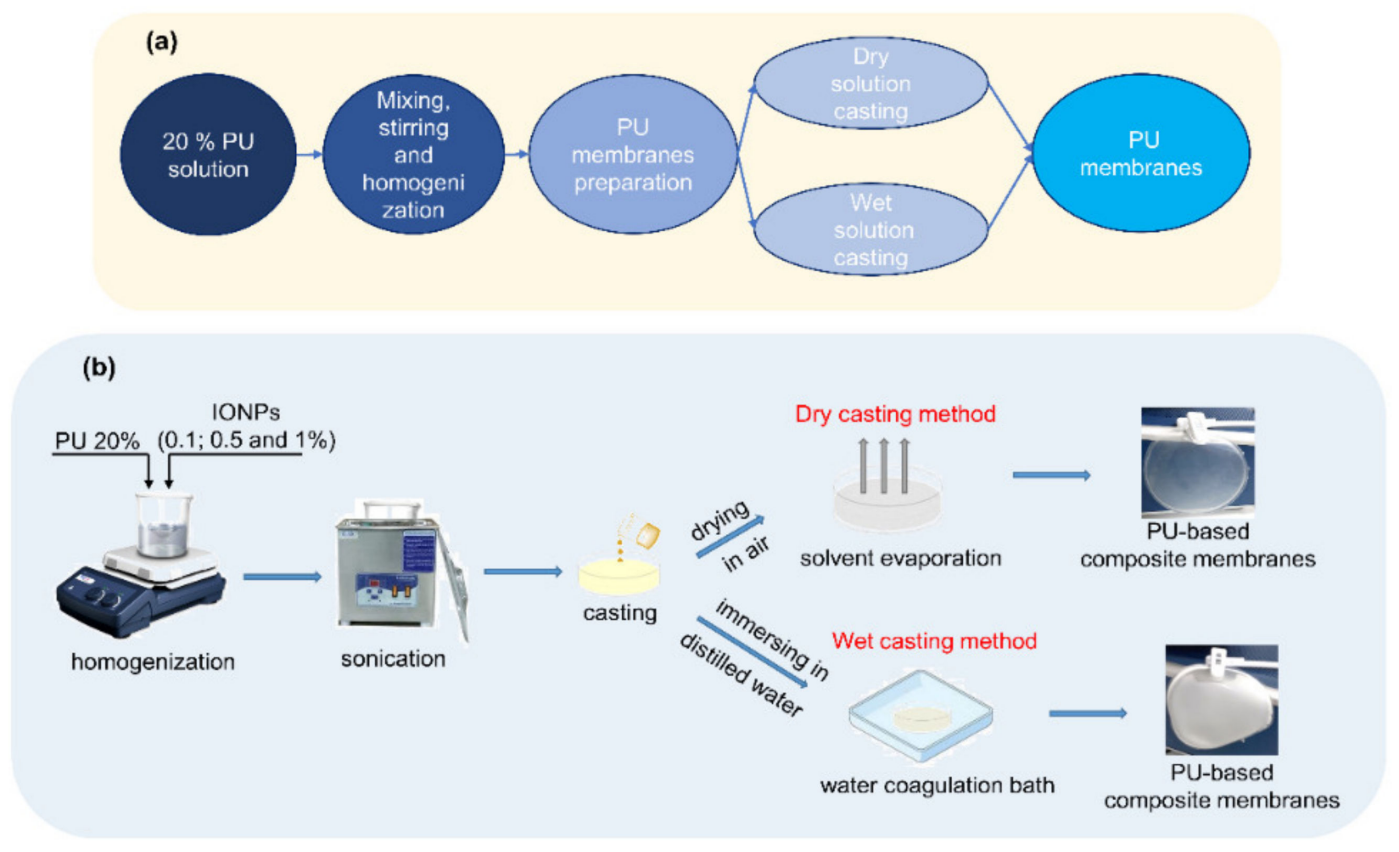
Illustration of membrane preparation methods of (**a**) polyester or polyether urethanes and (**b**) polyurethane-based composites.

A representative preparation procedure of polyester or polyether urethanes membranes and their composites is depicted in Scheme 2. The dried PESU and PETU were redissolved in DMF (20 wt.%) and well-mixed for a better homogenization (2 h at 1000 rpm). The solutions were degassed under vacuum (10–15 mmHg). The membranes were prepared by varying the preparation process using two types of methods: dry and wet solution-casting methods (Scheme 2a). In the former method, the membranes are made by pouring casting solutions onto glass plates and leaving them to evaporate the solvent at room temperature for 48 h. Then, the membranes were dried at room temperature and low pressure (1–2 mmHg) for another 24 h. In the second method, the PU solutions were cast onto glass plates using a doctor blade with a gap of 0.6 mm. The cast films were subsequently immersed in a distilled water bath at 45 °C and kept for 24 h to be sure of the removal of the solvent from the produced membranes. The resulting membranes were intensively washed with distilled water and dried at room temperature and low pressure (1–2 mmHg). The PU-based composites were prepared by blending 20 wt.% PU solution with different percentages of iron oxide nanoparticles (IONPs): 0.1, 0.5, and 1 wt.%. (Scheme 2b). The dispersions were stirred vigorously for 2 h and then were ultrasonicated for 4 h to obtain stable and homogeneous suspensions. The composite membranes were prepared following the two casting methods listed above.

### 2.4. Characterization Methods

A Verios G4 UC scanning electron microscope (Thermo Scientific, Waltham, MA, USA) equipped with an energy-dispersive X-ray spectroscopy analyzer (Octane Elect Super SDD detector (AMETEK, Tokyo, Japan)) was employed to investigate the membranes’ morphology. All the membrane samples were sputtered before image acquisition, with a thin layer of 10 nm platinum using a Leica EM ACE 200 Sputter Coater (Leica Microsystems, Vienna, Austria) to enhance the conductivity. 

The surface topography of the samples was investigated by using a high-sensitivity Tencor Alpha-Step D-500 (KLA Tencor Corporation, Milpitas, CA, USA) stylus profilometer, which provides accurate surface-metrology measurements including step heights and roughness. The device measured the roughness parameters with a recording speed of 0.10 mm/s and a filtration interval of 0.060 mm. The measurements were performed at three different points for each type of sample. The average roughness (Ra) was calculated as representative roughness information. 

Static contact angles were measured using a CAM 101 Optical Contact Angle Instrument (KSV Instruments, Helsinki, Finland) equipped with an optical system (charge couple device—CCD) connected to a computer. Around 1 µL of test liquids (deionized water, ethylene glycol, and di-iodomethane) was dropped on the membrane surfaces with a Hamilton syringe, and the image was immediately sent via the CCD camera to the computer for analysis. To minimize the experimental error, the contact angle was measured at five random locations for each sample. All the measurements were performed at room temperature. The surface energy parameters were estimated using the method developed by Owens, Wendt, Rabel, and Kaelble [38], based on the contact angle values.

Water vapor permeability (WVP) tests were conducted according to ASTM E96 standard, using the water cup method, the most common laboratory technique for the determination of low-to-moderate permeability in porous materials [39]. According to this procedure, a cylindrical cup filled with 50 mL of distilled water was covered by the tested membranes and placed in a chamber under controllable conditions of humidity (50%) and temperature (37 °C). The initial weight of the cups after equilibration 0.5 h in the controllable chamber was measured and compared to their weight at different periods of time for 24 h. The results of WVP were calculated using the following formula:

WVP = G⁄(t × A)(1)

where G is the water vapor weight change in grams, t is the time in hours, and A is the permeation area in cm^2^. The WVP was calculated from the steady-state region of the water-losses’ time curves. The examined membrane area was A = 10 cm^2^. Duplicate samples were used for each experiment, and data are presented as mean ± standard deviation. The membrane thickness was measured by a digital thickness gauge (Klass Messzeuge, Kaiserslautern, Germany) for all the samples at six different positions, and the arithmetic averages were in the range of approximately 0.2 mm.

The tensile tests of the samples were performed using a universal mechanical testing instrument (Instron-5567, Instron, Norwood, MA, USA) at room temperature and 60% relative humidity. All the specimens were cut into standard dog shapes with a length of 40 mm, a width of 5 mm, and a thickness of 0.10–0.15 mm. The mechanical properties were obtained from the stress–strain curves and averaged for five specimens.

Attenuated total reflection Fourier-transform infrared (ATR-FTIR) spectra were recorded using a Bruker LUMOS FTIR microscope spectrometer (Bruker Corporation, Karlsruhe, Germany) equipped with an ATR reflection module (Attenuated Total Reflection) and a diamond crystal. The spectra were collected in the range 500–4000 cm^−1^, and the measurements were made by averaging over 64 scans at a resolution of 2 cm^−1^. All the spectra were recorded at room temperature.

The dielectric measurements were performed using an Alpha-N Frequency Analyzer (Novocontrol GmBH, Montabaur, Germany) and a Rhode–Schwartz NVR Network Analyzer (Milpitas, CA, USA), which worked in the frequency range of 20 kHz ÷ 8 GHz, with the impedance between 0.1 Ω and 10 kΩ and a tan (δ) accuracy of >3×10^−2^. The temperature was controlled by using a QUATRO-Power temperature control unit (Novocontrol GmBH, Montabaur, Germany) with an accuracy of 0.01 °C and stabilization with an accuracy of 0.1 °C. WinDETA 5.81/WinFIT software packages (Novocontrol GmBH, Montabaur, Germany) were used for the measurement, calibration, and analysis of the samples. The dielectric characteristics as a function of frequency were studied at the temperature of 25 °C. 

The magnetic properties were evaluated on an MPMS3 (7 T) SQUID vibrating-sample magnetometer (VSM) operated in DC mode (Lake Shore Cryotronics, Woburn, MA, USA), at ambient temperature (25 °C). The samples were weighed and then placed inside a capsule. The magnetization cycles were performed between −30 and 30 kOe.

The cytotoxicity tests were performed using MCF 7 epithelial cell line (purchased from the European Collection of Cell Cultures (ECACC)) at a concentration of 20 × 10^4^ cells/well. The metabolic activity of cells after 48 and 72 h of treatment was determined using the 3-(4,5-dimethylthiazol-2-yl)-2,5-diphenyltetrazolium bromide (MTT) assay, as described before [13] and in accordance with the instructions of the manufacturer. Briefly, after the addition of the MTT solution (0.25 mg/mL), the samples were incubated for 3 h in the dark at 37 °C. The purple formazan crystals were then dissolved in isopropanol, and the absorbance was read at 570 nm. The amount of formazan was correlated with the number of viable cells. The cell viability was normalized to the epithelial cells cultured in the media with negative control (without material). The experiments were performed in triplicate, and the results are presented as mean ± standard deviation (SD). Fluorescence and phase-contrast microscopy were used to examine the morphology of the cells after 72 h of direct contact with the cells. The cells were fixed with an aqueous solution of formaldehyde and stained with 2-(4-amidinophenyl)-1H-indole-6-carboxamidine (DAPI) for nuclei observation. The blue fluorescence of the cell nuclei was detected using a 358/461 nm excitation/emission filter with a Leica DMIL inverted microscope (Leica, Wetzlar, Germany).

## 3. Results and Discussion

### 3.1. Preliminary Studies for the Selection of Conditions for Membranes’ Preparation

Membrane structures and properties are closely related to the mechanism of the membrane-formation process. Slight variations either in formulation recipes or manufacturing parameters can greatly influence membrane performance. In addition to these, the selection of a polymer as the membrane material is essential for the practical application of the membranes. Generally, the polymers used for membrane fabrication must be highly soluble in some common solvents; resistant to chemical, thermal, and mechanical stresses; and less susceptible to fouling [40].

To this end, in the first stage, a series of polyester/polyether-urethane-based membranes (denoted PESU/PETU) were prepared, and the influence of various parameters was explored in order to select the optimal formation conditions of the membranes. The effects of the parameters including the polyol’s nature used in the synthesis, preparation methods, and addition of additive agents in the polymer solution on the manufacturing and morphology of the PU-based membrane were first assessed. 

Initially, two membranes based on PESU and PETU were fabricated using both dry and wet solution casting methods to assess the effect of the polyols’ nature used in the PU synthesis. In the dry solution casting method, phase separation occurs due to the decrease in the polymer solubility during the evaporation of the solvent. Thus, to induce phase inversion, this process depends on the modification of the thermodynamic state of the polymer solution [41]. The wet solution casting method, also known as the phase inversion method, is considered one of the most versatile and reproducible membrane preparation methods and can be achieved through non-solvent-induced phase separation. The counter diffusion between solvent (DMF) and non-solvent (water) leads to phase instability in the solution, which results in the separation of the phases to form a membrane, most often with an asymmetric structure [42]. Moreover, the structure and morphology of such membrane are formed as a result of the interplay of mass transfer and phase separation [43]. In our case, the porous PU-based membranes were formed after precipitation into a water (non-solvent for PU) coagulation bath. This method was extensively used for the development of porous membranes [44,45] in order to control the morphology of the membranes.

**Figure 1:**
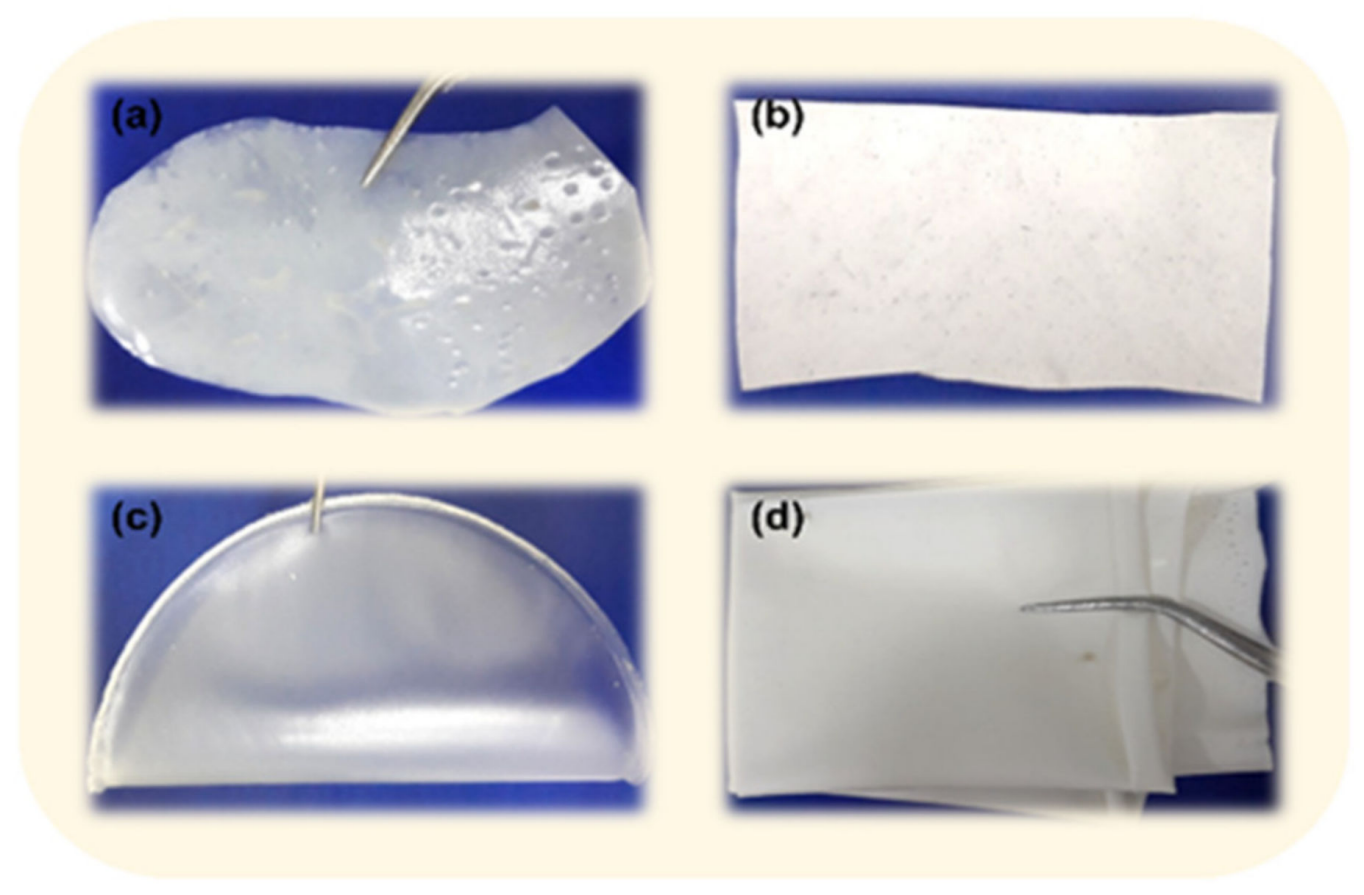
The effect of polyols’ nature on the preparation of the final membranes: (**a**) PESU-based membrane prepared by using dry solution casting method; (**b**) PESU-based membrane prepared by wet solution casting method; (**c**) PETU-based membrane prepared by dry solution casting method; (**d**) PETU-based membrane prepared by wet solution casting method.

The prepared membranes were shown in Figure 1. It is observed that the PESU-based membrane presented macrophase separation due to both preparation methods (Figure 1a,b). These defects are due to the irregular packing and aggregation of the polyester urethane chains and incomplete coalescence of polymer molecules in the layers [43].

The corresponding photographs of PETU-based membranes (Figure 1c,d) indicated that these membranes exhibited certain advantages over PESU-based membranes. Generally, the macroscopic phase equilibrium between the different components of the polymer, solvent, and non-solvent gives the thermodynamics of the membrane formation, which is further dictated by the choice of the polymer (its molecular architecture, the composition of the block component, etc.) [45]. Thus, PETU-based membranes presented good equilibrium thermodynamics among polymer–polymer and polymer–solvent molecules interactions and, thus, compatibility, resulting in a stable and homogeneous system. As observed in Figure 1c,d, the obtained PETU-based membranes using both preparation methods had a smooth and flat surface without irregularities.

**Figure 2:**
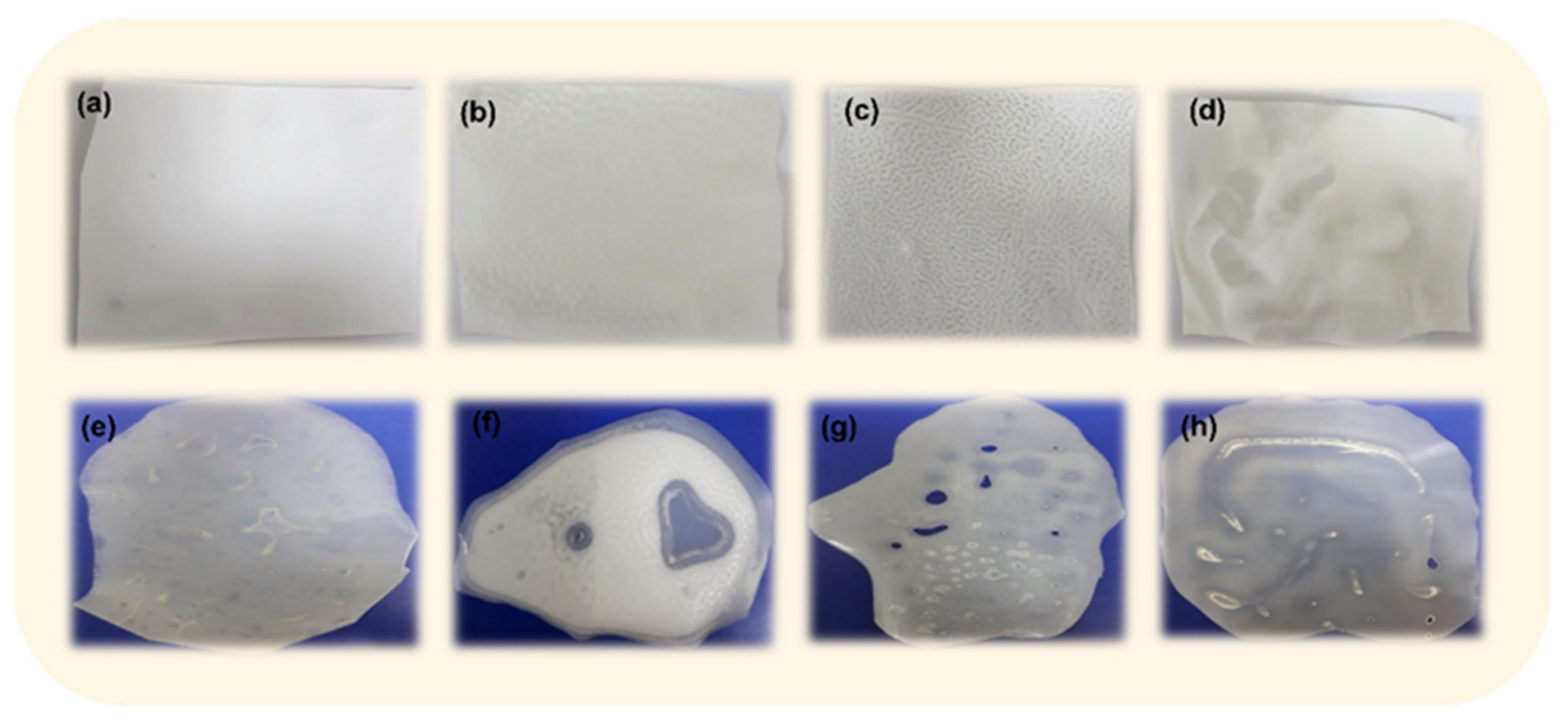
The effect of the additive addition in PESU-based solution before membrane preparation in wet solution casting: (**a**) without additive, (**b**) with 1%, (**c**) 2%, and (**d**) 5% of L-31; and in dry solution casting: (**e**) without additive, (**f**) with 1%, (**g**) 2%, and (**h**) 5% of L-31.

**Figure 3:**
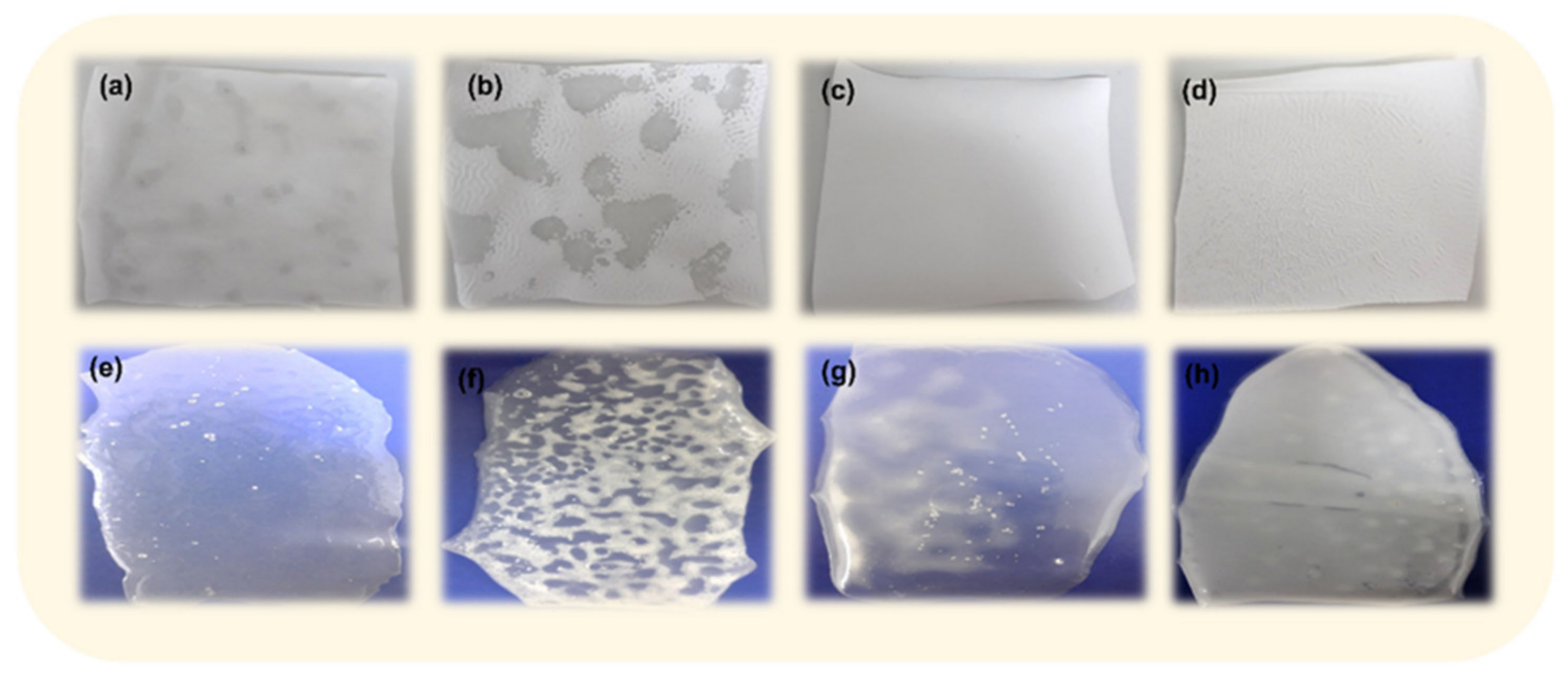
The effect of the additive addition in PETU-based solution before membrane preparation in wet solution casting: (**a**) without additive, (**b**) with 1%, (**c**) 2%, and (**d**) 5% of L-31; and in dry solution casting: (**e**) without additive, (**f**) with 1%, (**g**) 2%, and (**h**) 5% of L-31.

Another factor affecting the manufacturing process is the addition of some agents/additives in the polymer solution before membrane preparation to improve the morphological and physico-chemical properties and, in our case, to also improve the dispersibility of the IONPs in the PU matrices. The most common agents used for this purpose are hydrophilic polymers (polyvinylpyrrolidone and polyethylene glycol), various surfactants (Tween 20, Span-20, and Span-80), and nanoparticles (carbon nanotubes, silica nanoparticles, and inorganic nanoparticles) [40,44]. This means that the final membrane morphology could be tailored by the amount of these agents, which leads to optimized membrane properties. However, to evaluate the influence of a certain additive on the PU-membrane-preparation method, we used a dihydroxy-functional oligomer-poly(ethylene glycol)-block-poly(propylene glycol)-block-poly(ethylene glycol) with Mn of 1100 Da, known as Pluronic L-31, which is a neutral amphiphilic surfactant utilized for many purposes, especially in different biomedical applications [46,47]. We choose this additive because it is a surface-active material with properties that are dependent on the hydrophilic/hydrophobic ratio and has a strong pore-forming ability, leading to the improved surface morphology of the membranes [40]. Moreover, this surfactant could also improve the stability of the IONPs in the PU matrices. Thus, we prepared PESU- and PETU-based membranes with different concentrations of L-31 (1, 2, and 5 wt.%). The samples were obtained by both dry and wet solution casting methods, and their photographs are illustrated in Figure 2 and Figure 3.

The addition of L-31 in PESU-based membranes was leading to the phase separation of the structures, for both the wet (Figure 2b–d) and dry (Figure 2f–h) casting methods, for all the embedded concentrations. Thus, the PESU-based membranes embedded with different concentrations of L-31 prepared in wet conditions by solvent casting method are non-homogenous, with contractive surfaces (Figure 2d) and a “snake skin” look (Figure 2b,c). The membranes prepared in dry conditions, by evaporation of the solvent, also present phase separation. A very nice example was the “heart-shaped hollow” observed in the PESU-based membrane prepared with 2% L-31 (Figure 2f). This separation between molecular chains comes from the incompatibility and weak interaction between L-31 and the polyester urethane structures. 

In conclusion, for both casting methods, the addition of L-31 in the PESU-based membranes showed a negative effect on membrane preparation, so these membranes were not further investigated.

The introduction of L-31 units into the PETU-based matrix resulted in an increase in the compatibility between the two structures, but not for all the concentrations of added surfactant (Figure 3). Thus, the PETU-based membranes prepared in wet conditions by the solvent casting method presented different morphologies. At 1% of L-31 (Figure 3b), the membrane presents a non-homogeneous structure with phase-separated domains and shrinkage. When the additive concentration increases, the overall hydrophilicity of the polymer is enhanced, and, hence, the solvent-nonsolvent exchange becomes faster, facilitating instantaneous demixing. The optimum additive concentration for the preparation of the uniform membranes was observed at 2% of L-31 (Figure 3c). Further, when the additive concentration exceeded a certain amount (5% of L-31), the overall viscosity became so high that the diffusion of nonsolvent was restricted, leading to a shrinking surface (Figure 3d). 

The PETU-based membranes prepared in dry conditions by evaporation of the solvent also present phase separation (Figure 3e–h). However, the best-prepared membrane was also the one in which 2% L-31 was added (Figure 3g), even if it showed some crystallization points.

Taking these aspects into consideration, PETU-based solution with 2 wt.% of L-31 was chosen as the optimized blend to prepare membranes for further study.

**Figure 4:**
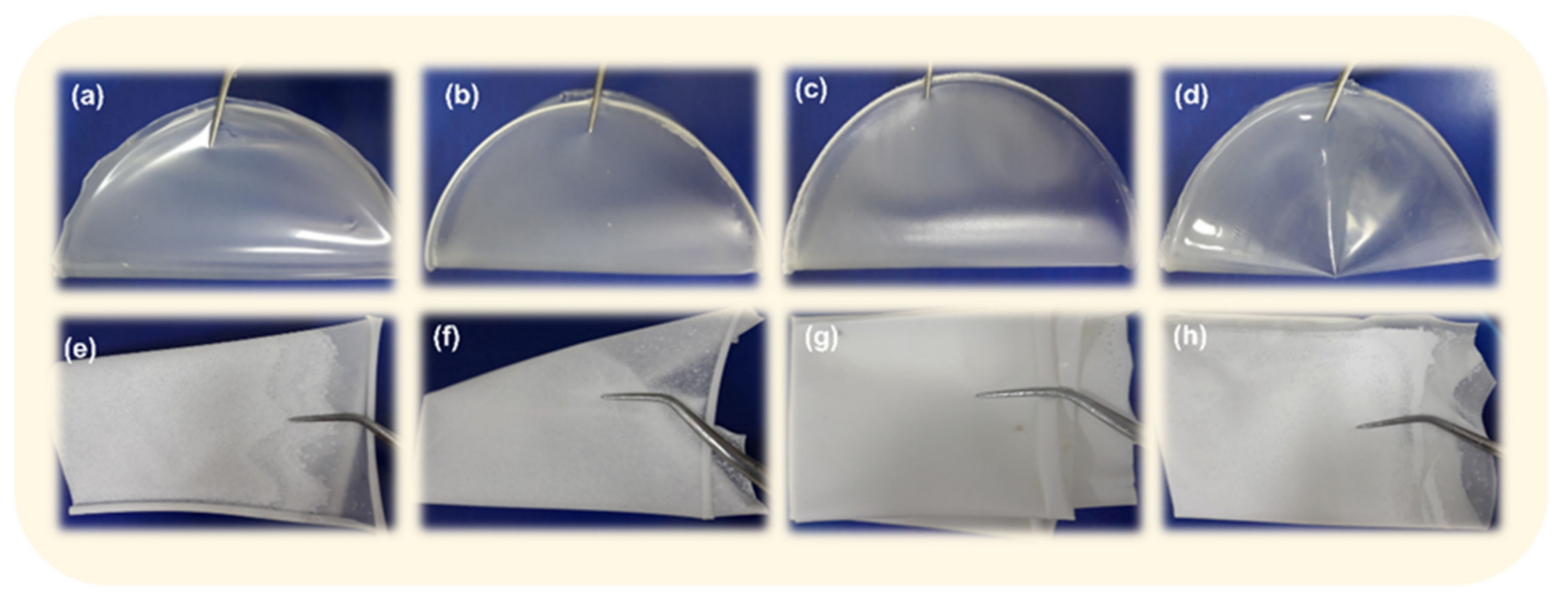
The effect of the casting temperature on the PETU-based membranes prepared by dry solution casting method at: (**a**) room temperature (25 °C), (**b**) 40 °C, (**c**) 60 °C, and (**d**) 80 °C; and in wet solution casting method: (**e**) direct solvent casting at 45 °C, (**f**) 15 min, (**g**) 30 min, and (**h**) 60 min air precoagulation and then immersing in a water bath at 45 °C.

The influence of film-casting conditions such as dry temperature, precoagulation time, and coagulation bath temperature was also investigated. Figure 4 shows the PETU-based membranes prepared in different conditions by the dry and wet methods. Thus, after drying under various temperatures (room temperature, 40, 60, and 80 °C) (Figure 4a–d), the obtained membranes exhibited very small differences. According to the literature [48], the increase in the drying temperature may result in a strong increase in the polymer segmental motions, leading to an exponential increase in the molecular diffusion rate of the solvent. This caused the drying of the membranes at different rates, but this has no effect on the final membrane’s appearance. In these conditions, the variation of the temperature had the lowest influence on the membrane preparation due to the evaporation rate of the solvent used in the preparation of the solutions.

When the membranes were prepared in wet conditions, the differences are obvious to observe. The membranes changed from a nodular and non-homogeneous (Figure 4e,f,h) morphology to a smooth and uniform (Figure 4g) morphology. Therefore, in the phase inversion casting technique, the membrane layers are formed immediately when immersed in the coagulation bath and depend on the temperature of the non-solvent bath and also on the amount of time of the coagulation before and after immersion. The influence of the temperature of the solution bath was previously reported in another of our works [49], which was chosen as 45 °C, for was the optimum temperature for the formation of uniform, sponge-like pores.

When the membrane was prepared by direct immersion in the non-solvent (water) coagulation bath, it presented a phase-separated structure with beads and a non-homogeneity of the layer (Figure 4e). This phenomenon is due to the rapid solvent out-flow rate, which limits the conformational and configurational rearrangement of the macromolecular chains, contributing to the non-homogeneity of the membrane. To mitigate this aspect, evaporation of the volatile solvent, before immersing it in the coagulation bath, is a common treatment to improve the membrane structure. The evaporation step, which we called the precoagulation step, prevents the shrinkage of the membrane during immersion and also suppresses the formation of macrovoids [40]. Moreover, the time period that occurs between the immersion of the polymer solution into the coagulation bath and the start of liquid–liquid demixing strongly influences the morphology of the resulting membranes [40]. In this way, it is possible to prepare membranes with a uniform distribution of porosity. In order to obtain smooth and uniform membranes, we studied the influence of the precoagulation time before immersion in a water coagulation bath. It is well-known that the thin outer layer, which is formed during dry evaporation, acts as a resistive barrier between the water from the coagulation bath and the interior of the membrane, lowering the coagulation rate [50]. For example, the literature studies revealed that the diffusion coefficient of the outer layer is much lower than that of the bulk membranes [43]. Thus, in our study, the best performance was achieved for the membrane that was precoagulated for 30 min in air and then immersed in a water bath at 45 °C, when a favorable equilibrium between the out-flow of the solvent (DMF) and the in-flow of water takes place.

**Figure 5:**
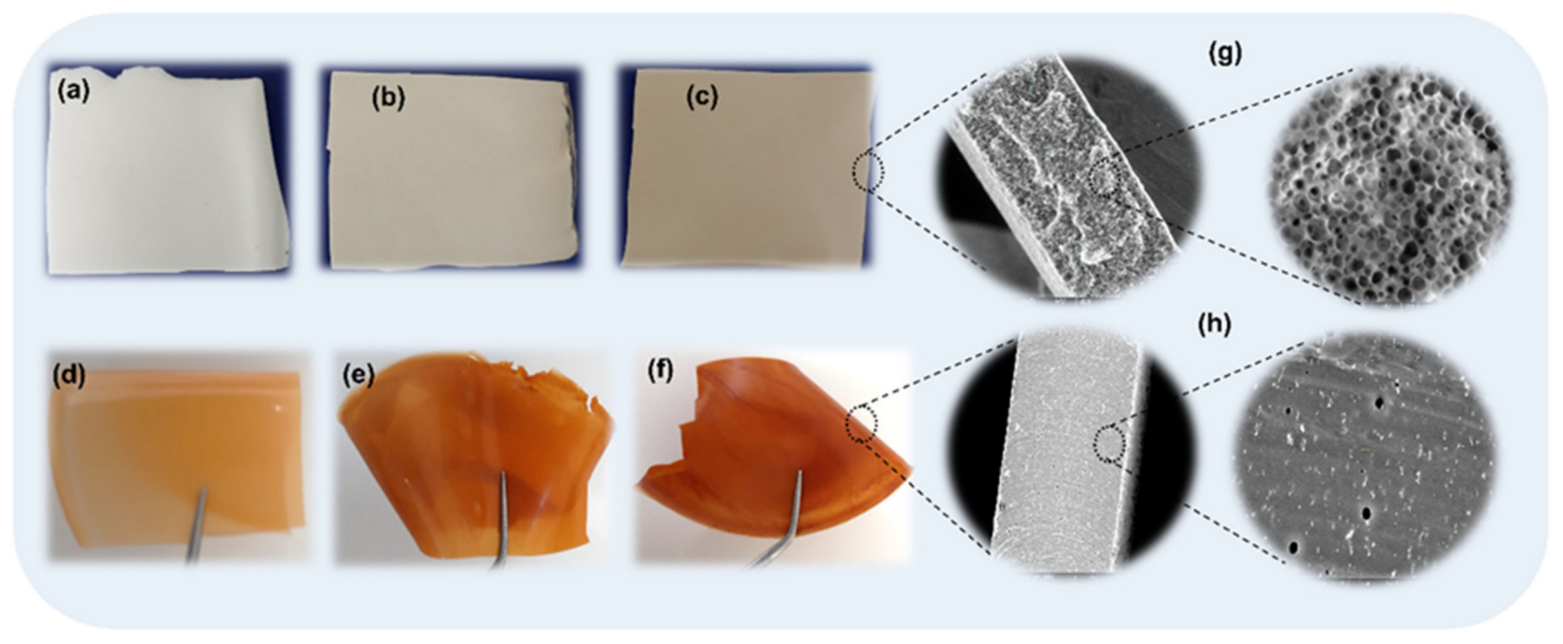
Illustration of composite membranes prepared by wet solution casting method (Pw), with (**a**) 0.1%, (**b**) 0.5%, and (**c**) 1% IONPs content, and in dry solution casting method (Pd) with (**d**) 0.1%, (**e**) 0.5%, and (**f**) 1% IONPs content. Cross-sectional visualization of membranes by SEM (scale bare 200 µm with magnification 100× and 20 µm with magnification 5000×, respectively) corresponding to the 1% IONPs content, prepared by wet (**g**) and dry (**h**) solution casting methods.

Taking into account the aforementioned preliminary results, composite membranes with the optimum conditions, as far as the membrane structure is concerned, were prepared. The conditions included 20 wt.% PETU-based solution in DMF, addition of 2 wt.% triblock copolymer Pluronic L-31, casting in both dry (room temperature air) and wet (distilled water bath at 45 °C with 30 min precoagulation time) solution methods. Keeping these conditions, stable membranes without defects could be prepared. These blank samples were denoted in the following section as Pd and Pw, respectively. To study the influence of the IONPs content on the properties of these membranes, we chose to formulate the membranes at concentrations of 0.1, 0.5, and 1 wt.% of IONPs (Fe_2_O_3_). The composite samples were denoted as Pd-0.1, Pd-0.5, and Pd-1, respectively, for the membranes prepared in dry casting conditions and as Pw-0.1, Pw-0.5, and Pw-1, respectively, for the membranes prepared in wet conditions. The prepared membranes following both casting methods are illustrated in Figure 5. 

It is well-known from the literature [2] that the membranes’ preparation method has a great influence on their morphology. Therefore, it was observed that after the wet casting method, porous membranes were obtained (Figure 5a–c), instead of the dense, non-porous structures prepared by dry casting (Figure 5d–f), as were revealed by the cross-section visualization of the membranes (Figure 5g,h) by SEM. Thus, the preparation method plays a crucial role in the final structure of the membranes.

The membranes prepared by the wet solution casting method present a cellular sponge structure due to the liquid–liquid demixing mechanism (Figure 5g). As previously reported, the addition of 2% of L-31 in the polyurethane structure led to the slowdown of the solvent exchange rate when the membranes were immersed in the coagulation water bath, governing the overall porosity. Moreover, the addition of IONPs increases the interaction and self-organization of the swelled macromolecular chains, leading to interconnected structures. Thus, the porous membranes feature uniform and well-contoured pores with a mean diameter of 2.9 µm. 

During solvent evaporation, as in the case of the membranes prepared in dry conditions, a dense and non-porous structure results, as revealed by the SEM cross-section image (Figure 5h). However, some macrovoids were observed at the cross-section of the membranes, probably due to the formation of gas bubbles during the drying process.

### 3.2. Characterization of Magnetic Polyurethane-Based Composite Membranes

To evaluate the performance of the polyurethane-based composites, the morphological, physico-chemical, mechanical, dielectric, and magnetic properties; wettability; and some preliminary cytotoxicities were studied.

#### 3.2.1. Surface Morphology Investigations

##### SEM Analysis of the Surfaces

**Figure 6:**
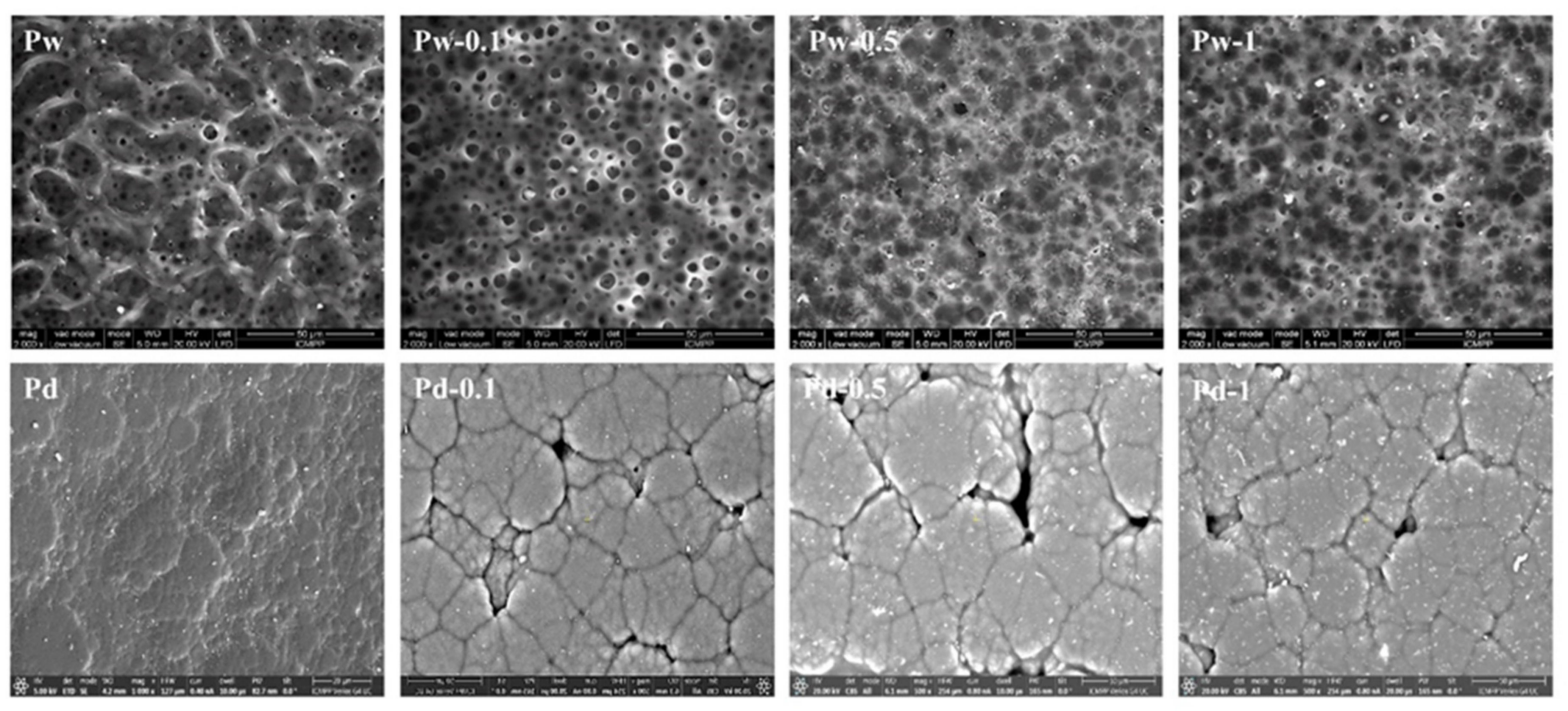
SEM analysis of membrane surfaces prepared by wet (Pw) and dry (Pd) solution casting methods and embedded with different concentrations of IONPs (0.1, 0.5, and 1 wt.%).

Since membrane performances directly depend on their morphology (pore size and distribution), morphology control is the key factor in membrane fabrication. To characterize and visualize the surface morphology, an SEM investigation was carried out. The characteristic SEM images corresponding to the PU-based composites embedded with different concentrations of IONPs prepared by the wet and dry solution casting methods are illustrated in Figure 6.

The porous membrane without IONPs (Pw) obtained by the wet solution casting method (Figure 6) presented a uniform distribution of interconnected large and small pores, with diameters between 4 and 20 µm. When the IONPs were added (Pw-0.1, Pw-0.5, and Pw-1, respectively), the morphology completely changed due to the different mechanisms of membrane formation. As the literature reported [51], at low concentrations, the IONPs act as nucleating agents, generating a uniform distribution of pores at the surface. The increase in IONPs concentration causes a decrease in the porosity due to the solution viscosity increasing, which decreases the exchange rate [52]. Thereby, the increase in the solution viscosity delayed the phase inversion phenomenon, leading to a decrease in the membrane pore size. In this case, the morphology of the membranes mainly depends on the thermodynamics and kinetics of the phase inversion process [53]. Moreover, by increasing the concentration of IONPs, the number of hydrogen and coordination bonds with the polyurethane structure increased, and, accordingly, this led to a decrease in the membrane pore size.

On the other hand, the literature indicated that at higher concentrations of filler in the matrix, a very small particle size for the filler should be used in order to prevent their sedimentation and agglomeration during membrane formation [54,55]. Therefore, in this study we used ultrafine magnetic nanoparticles (<50 nm) to reduce the sedimentation rate in the membrane-preparation process and to increase the number of inter- and intramolecular interactions.

As can be observed, for the membranes prepared by the dry solvent casting method (Pd), the addition of IONPs (Pd-0.1, Pd-0.5, and Pd-1, respectively) led to the appearance of cracks at the surface after drying, due to the existence of a strong tension between the polymer molecules and IONPs. During the drying process, the solvent (DMF) is slowly evacuated from the matrix, and the polymeric network has enough time to collate and separate the phases, resulting in a dense and non-porous structure for the matrix.

One of the major challenges in the membrane field is to obtain a homogeneous morphology of the prepared materials. Therefore, optimizing the morphology of a certain structure will enhance the physical and chemical properties of the modified material. Taking into consideration the visual illustration of the membranes (Figure 5) and the SEM analysis of the surfaces (Figure 6), which revealed that the membranes prepared in wet conditions showed a good homogenization of the IONPs (Figure 5a–c) and a nice surface porous structure (Figure 6, Pw), instead of the non-homogeneity (Figure 5d–f) and dense structures with small cracks on the surfaces (Figure 6, Pd) of the membranes obtained under dry conditions, we decided to further characterize only these porous membranes prepared by the wet solution casting method. Moreover, we want to develop an external device based on this PU-based membrane, which will enhance the diagnostic capabilities of magnetic resonance imaging investigations, so the membrane should have some porosity in order to achieve diffusivity and permeability by the molecules.

##### Surface Roughness

**Figure 7:**
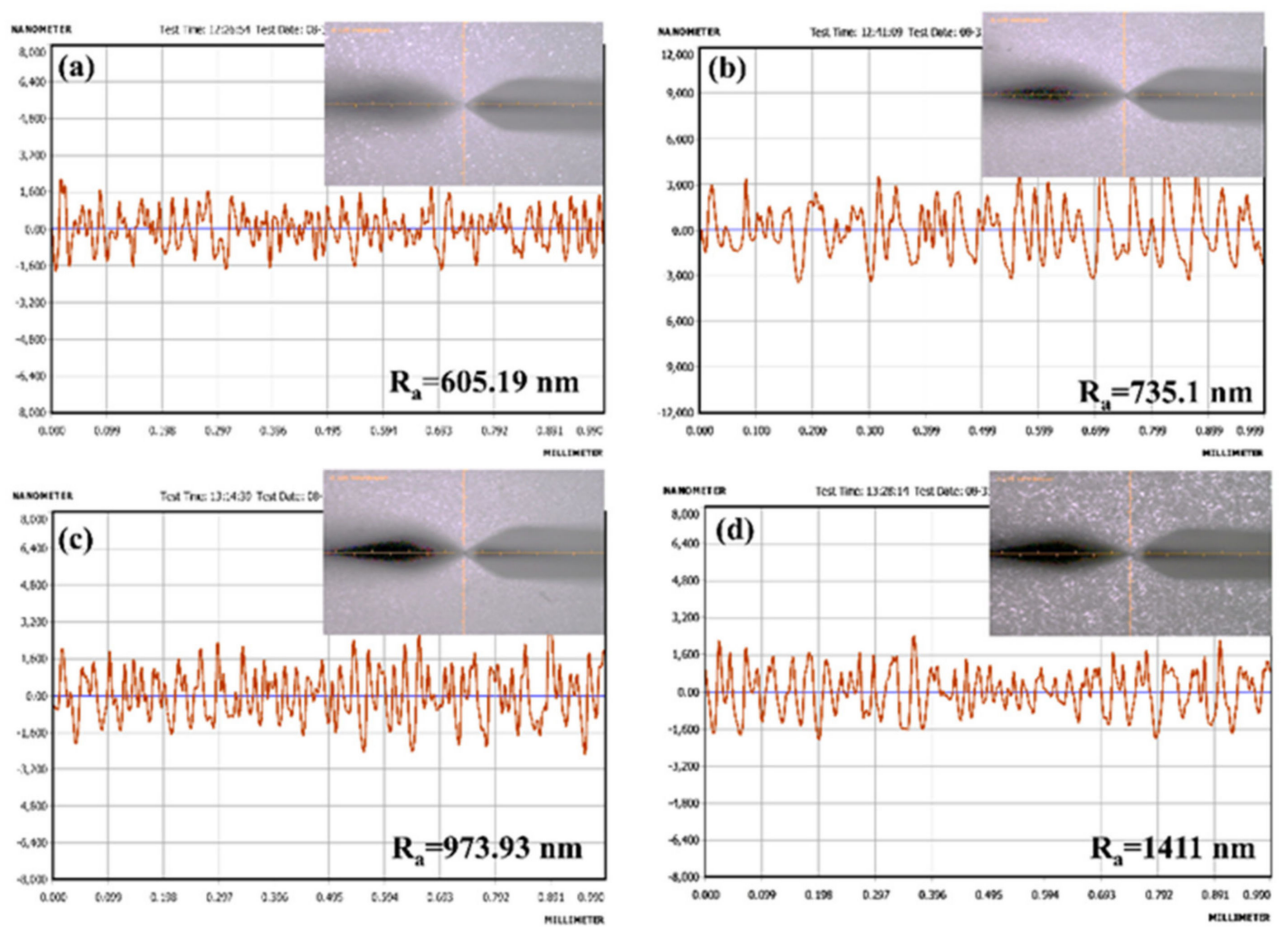
Microscope images and profilometric histogram of polyurethane-based composite membranes prepared in wet conditions with different amounts of IONPs: (**a**) 0%, (**b**) 0.1%, (**c**) 0.5%, and (**d**) 1%.

Surface analysis was also used to investigate the surface roughness of the membranes before and after the incorporation of IONPs. Profilometry is a quantitative technique known to reflect the irregularities of the surface profile of the material. The roughness parameter is one of the best parameters for comparing different membranes. Moreover, surface roughness is recognized as an important factor for cell interactions [56], especially for osteoblasts or chondrocytes [57], which govern material biocompatibility in part [58]. Thus, the arithmetic average of the roughness profile (Ra) was calculated as the average roughness of the surface-measured microscopic peaks and valleys. Therefore, the profilometric analysis of the surfaces confirms that the roughness of the membrane surface is dependent on the IONPs content (Figure 7). Thus, the Ra of the original membranes without IONPs (Pw) was found to be 605 nm. When the membranes were embedded with IONPs, an increase in Ra was observed up to 1411 nm for the sample with 1 wt.% (Pw-1), leading to a rough morphology at the membrane surface. The increased roughness could be attributed to the increase in the nanoparticles content and also to the change in the porosity of the membrane surfaces. The topographic images of the membrane surfaces, illustrated in the right corners of Figure 7, display the same tendency. These results corroborate well with those from the SEM evaluation, which suggested that the number of pores increases and their diameters become smaller, with the increase in the IONPs content. Moreover, these results are in agreement with other literature studies [59].

##### Surface Hydrophilicity and Surface Energy

**Figure 8:**
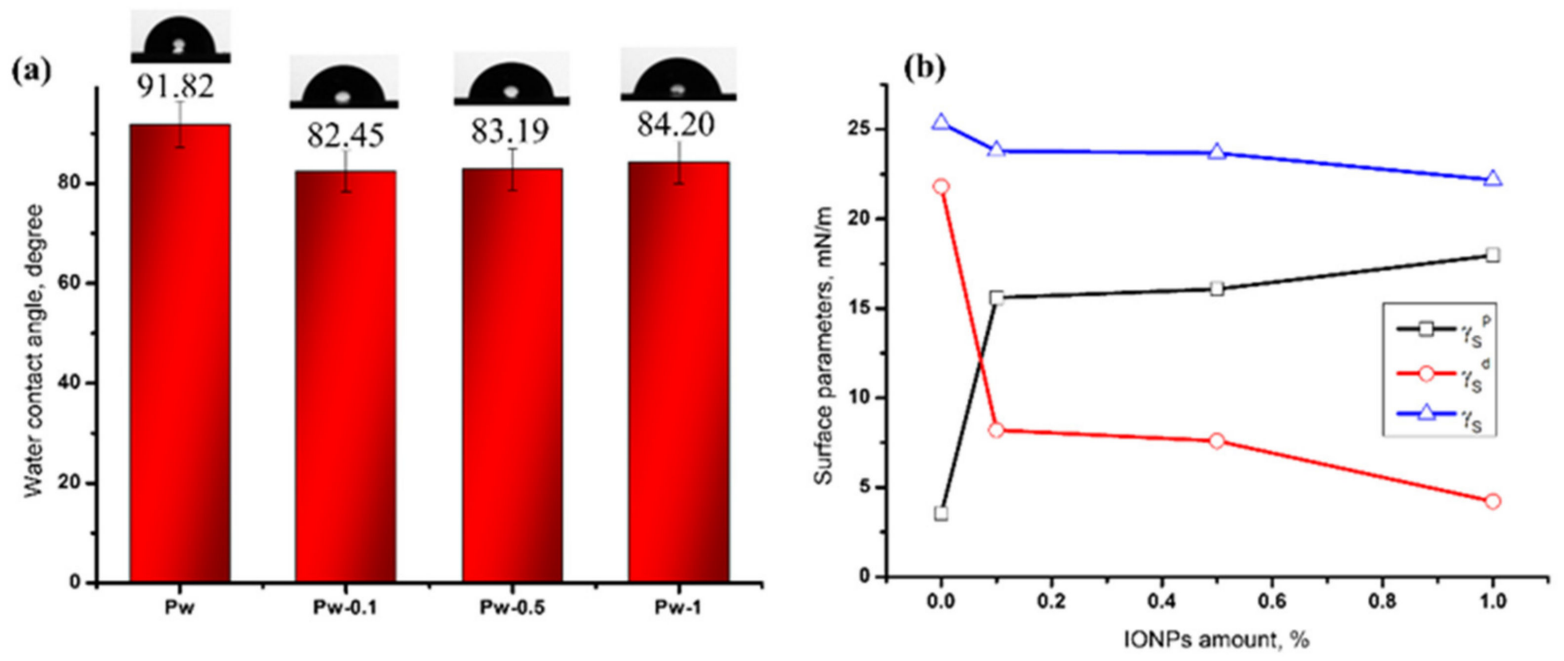
Variation of (**a**) water contact angles and (**b**) surface parameters with IONPs concentration for the PU-based magnetic composites.

To study the changes in the surface properties of PU-based composite membranes, related to the modification by the addition of IONPs, the sessile drop method for the measurement of the contact angles of water was used as one of the most common methods to indicate membrane hydrophilic–hydrophobic features. Moreover, the measurement of contact angle not only quantifies the interaction between the membrane surface and the liquid drops but also facilitates the understanding of the physical and chemical processes involved in the fabrication of the desired composite membrane surfaces. The obtained values of contact angles for the corresponding composite membranes are presented in Figure 8a. The water contact angle analysis shows the surface wettability of the prepared membranes. As shown in this figure, the contact angle of pure PU membranes (Pw) has a value of around 90°, indicating that this membrane has a hydrophobic surface. The addition of IONPs changed the hydrophobicity of the composite membrane surfaces, decreasing around 10°, up to 80°. Thus, the decrease in contact angle values indicated an increase in the hydrophilicity of the composite membranes. We believe that the introduction of iron oxide nanoparticles into the polyurethane matrix led to the establishment of hydrogen and some coordination bonds between the Fe_2_O_3_ complex and the N-H or C=O groups of the polyurethane structure. These types of interactions caused an improvement in the surface hydrophilicity.

The surface energy (γ_s_) and its components (γ_s_^p^, γ_s_^d^) are essential for understanding the mechanism of surface-based phenomena, which, in turn, are important in a variety of applications such as adhesion, coating, printing, and so on. Therefore, the wetting of a solid surface is one of the major concerns in large-scale industrial processes. Based on experimental contact angle values and using the mathematical method developed by Owens, Wendt, Rabel, and Kaelble [38], the surface parameters could be calculated. The variation of the calculated surface energy parameters is shown in Figure 8b. The results indicated that the addition of IONPs decreases the values of the overall surface free energy (γ_s_). If we are looking at the surface free energy parameters, it is observed that the polar components (γ_s_^p^) were significantly higher than the dispersive components (γ_s_^d^), which suggests that the dipole–dipole and hydrogen-bonding interactions are more substantial than the van der Waals interactions between the analyzed surfaces and liquid drops. Thus, the existence of polar and non-polar groups on the surface directly influenced the wettability property.

Surface properties represent the key requirements for the biomaterials, due to the fact that they determine the degree of biocompatibility and biofouling when interacting with biological systems. All the studies suggest that the membranes used mainly in the biomedical field should have a balanced distribution of hydrophilic and hydrophobic domains [60]. Overall, moderate surface wettability is more able to bind cells than highly hydrophobic or hydrophilic surfaces [61]. Moreover, biofouling is an important factor in the preparation of biomaterials, since the non-specific adsorption of undesired molecules, proteins, or other substances on the membrane surface can affect its functionality [1]. For example, in biosensor fabrication, the prevention of biofouling on the membrane surfaces is the key factor to improve sensitivity and selectivity. Moreover, the performance of a sensor depends on the electrode material, and, thus, membranes can be used to cover and modify the surface of the electrodes to improve their sensitivity.

##### Water Vapor Permeability

Generally, the permeability of polymeric materials is quantified by the amount of mass exchange between the material and the external environment. Therefore, the potential ability of a polymer matrix to sorb the permeant molecules and the ability of those molecules to diffuse through the polymeric material determine the transport of penetrant molecules through polymeric materials [62]. This transport is determined by different intrinsic (crystallinity, orientation of molecules, free volume, cohesive energy density of polymer, size and type of pores, etc.) and extrinsic factors (temperature and moisture conditions). Since the extrinsic factors are controlled, only the intrinsic factors affect the barrier properties. Hence, the water vapor permeability (WVP) of the PU-based composite membranes before and after the incorporation of IONPs was determined. Table 1 shows the loss of water vapor with time through the membrane and the calculation of WVP when the samples were incubated for 24 h in a conditioned room (37 °C and 50% humidity).

As seen in Table 1, the water vapor loss over time (G/t) increases with the increasing IONPs content. The R^2^ values are higher than 0.99, meaning that the linear regression fits the data points well. The WVP was calculated by dividing the slope values by the area of the cup mouth (A). The membrane with 1% IONPs (Pw-1) exhibited a higher WVP value (66.5 ± 1.1 g/m^2^h), while the pure membrane (Pw) displayed a much lower value of WVP (49.7 ± 0.9 g/m^2^h) within 24 h. The measurements of the WVP allow for quantifying the amount of water that diffuses through the membranes per unit area in time. Thus, the results suggest that the increase in the IONPs content resulted in increased WVP values, meaning that a large amount of water vapor is able to diffuse at a concentration of IONPs up to 1%. The main factors affecting the WVP are the porosity and hydrophilicity of the membranes [63,64]. Therefore, the WVP increase can be explained by the decrease in the diameter of pores and the increase in the number of pores with the addition of IONPs, as illustrated in SEM investigations. In addition, this increase may also be a result of the increase in hydrophilicity of the composite membranes, as observed in the contact angle measurements. As previously related, the introduction of IONPs into the PU matrix led to the establishment of hydrogen and some coordination bonds between the Fe_2_O_3_ complex and the N-H or C=O groups of the PU structure. These types of interactions determined the improvement of the hydrophilicity as well as the number (density) of the pores, even though the diameter of the pores decreased. Moreover, the introduction of IONPs did not block the pores of the membranes, and, therefore, these membranes could be able to develop devices with good breathability.

In conclusion, the information on the interaction of moisture with polymeric membranes is of fundamental importance for solving problems of material science, such as the choice of material for specific aims or predicting the behavior of materials when they are in contact with moisture during storage or application.

#### 3.2.2. ATR-FTIR Investigations

The IR spectra were recorded using a fully automated stand-alone FTIR microscope equipped with an ATR reflection module, which offers outstanding visual and spectral data quality. ATR-FTIR is a powerful tool to identify various types of chemical bonds in macromolecules, by producing an IR absorption spectrum that is like a molecular, specific fingerprint. Moreover, the microscopic visualization provides the opportunity to view the sample’s surface. Thus, infrared spectroscopy was applied to monitor the changes of the chemical compositions of the PU-based membranes before and after the addition of IONPs.

**Figure 9:**
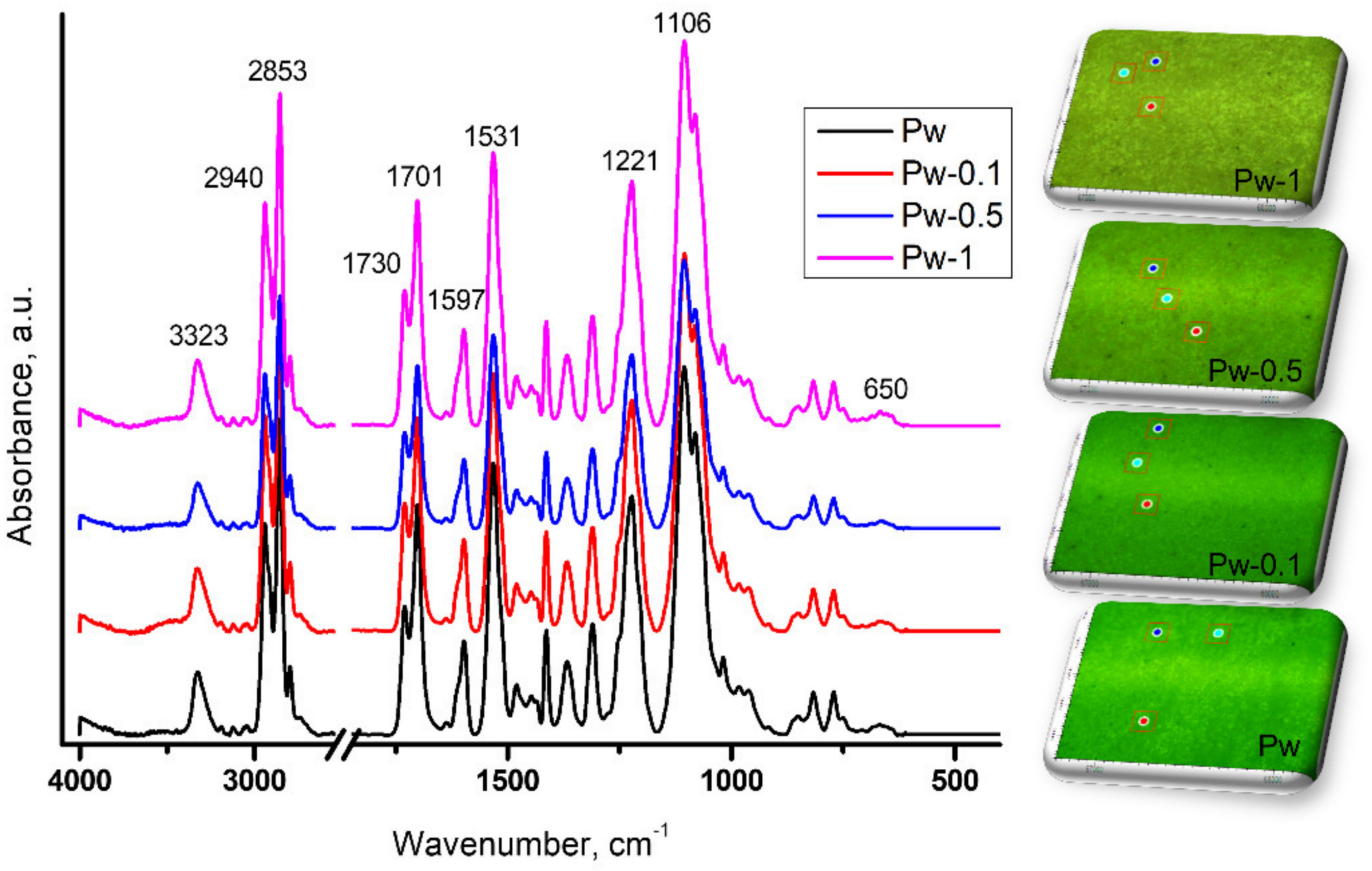
ATR-FTIR and corresponding microscope images of PU-based composite membranes prepared in wet conditions with different amounts of IONPs.

Figure 9 shows the ATR-FTIR spectra of membrane surfaces without (Pw) and with different concentrations of IONPs (Pw 0.1–1). The membrane without IONPs content (Pw) presents the characteristic peaks of the polyurethane structure. Thus, the bands associated with the stretching vibration of -NH, which overlap with those of the -OH groups, appear at 3322 cm^−1^. The bands at 2940 and 2853 cm^−1^ were assigned to the asymmetric and symmetric stretching vibration of the C–H bond in CH_2_ groups. At 1730 and 1701 cm^−1^ are the bands corresponding to the free and bonded C=O stretching vibration of the urethane groups, respectively. The stretching vibration of the C-C groups in the aromatic rings of MDI was observed at 1597 cm^−1^. The skeletal aliphatic C–C/aromatic hydrogen bending and aliphatic C–H rocking appeared at 1200–900 cm^−1^ [13].

Almost the same spectra were observed for the membranes with different IONPs content. The absence of the characteristic peaks of IONPs from the spectra (600–500 cm^−1^) may indicate its complete incorporation in the polymer matrix, without a significant contribution to the surfaces, due to the covering of the IONPs with polymer. Therefore, the bands characteristic of the polyurethane structure covered or enveloped the vibration of the iron oxide structure. This phenomenon is also due to the small number of nanoparticles embedded in the PU matrix.

Furthermore, according to Figure 9, the microscope observations highlighted different surface morphologies of the PU-based magnetic composite membranes, which were also pointed out by the SEM and profilometric evaluations.

#### 3.2.3. Tensile Tests

**Figure 10:**
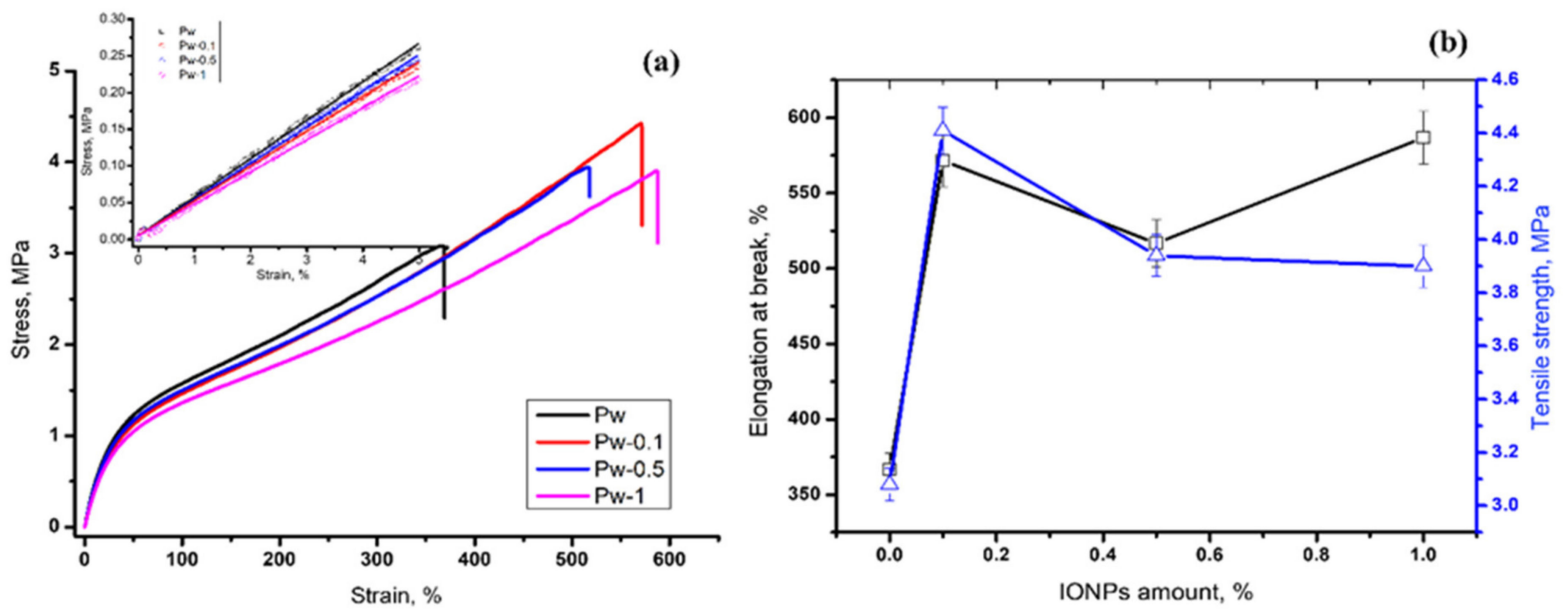
Tensile properties of PU-based composite membranes prepared in wet conditions with different amounts of IONPs: (**a**) stress–strain curves; inset: stress–strain curves in the elastic region; (**b**) variation of elongation at break and tensile strength as a function of the amount of IONPs.

To reveal the impacts of the IONPs content on the mechanical properties, tensile tests were performed. Figure 10a shows the stress–strain curves of the membranes without and with different concentrations of IONPs. As is observed, the stress–strain curves followed the same pattern for all of the investigated membranes. There is a short region of elastic deformation, followed by a plastic deformation region as the stress and the strain increase until failure. The first linear elastic zone of the curves is governed by the crystallization of soft domains and local reordering of the PU macromolecules, indicating a preferential reinforcement of the soft domains, instead of the hard segments observed in another study [16]. In the plastic deformation region, a breaking of the interconnecting network of hard domains appears until total failure.

Tensile strength is a measure of the material’s mechanical properties, since it is defined as the force required to break the specimen in a linear direction. Thus, it is defined as the ability to resist breaking under tensile stress, quantifying how much stress the material will hold before suffering permanent damage [10].

As shown in Figure 10b, the pure polyurethane membrane (Pw) displays an elongation at break of 366 MPa and a tensile strength of 3.08 MPa. By incorporating up to 1 wt.% IONPs into polyurethane, the membranes exhibit an increased elongation at break of 586 MPa and tensile strength of 3.9 MPa, respectively, for the sample Pw-1. This increase comes from the inter- and intramolecular interaction between the nanoparticles and polyurethane chains. Thus, the addition of IONPs causes an overall improvement in tensile properties compared to the neat polyurethane, achieving a reinforcement effect on the polyurethane matrix.

#### 3.2.4. Dielectric and Magnetic Properties

The dielectric and magnetic behaviors of the composite membranes are significantly influenced by the properties of the interfaces between the polyurethane matrix and magnetic nanoparticles [65]. In order to gain further insight into the bulk properties of the magnetic PU-based composite membranes, the dielectric and magnetic properties were studied.

**Figure 11:**
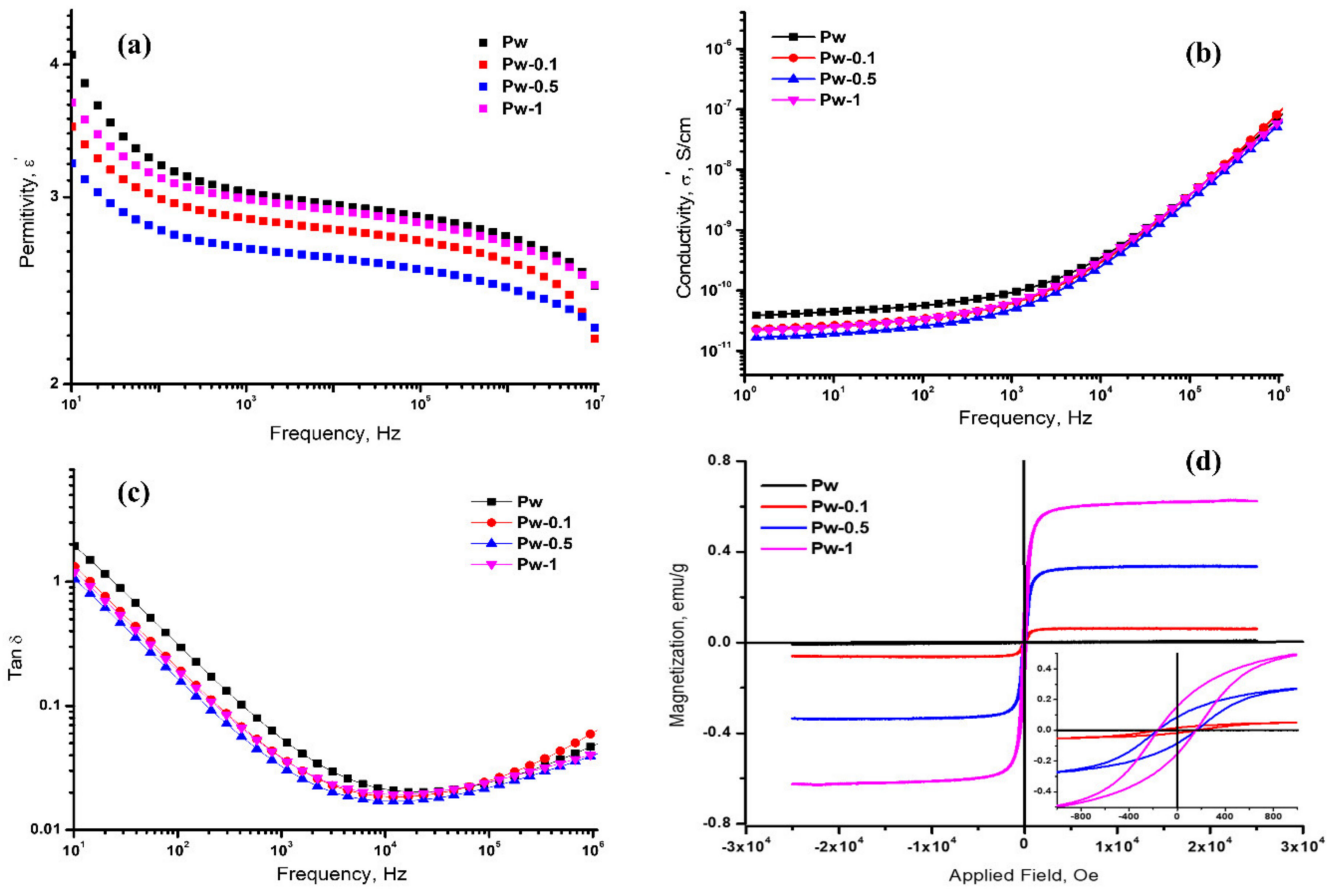
Frequency dependencies of (**a**) dielectric permittivity, (**b**) conductivity, (**c**) loss tangent, and (**d**) field-dependent magnetization curves of PU-based composite membranes prepared in wet conditions with different amounts of IONPs.

The dielectric properties of PU-based magnetic membranes were evaluated on the basis of permittivity, conductivity, loss tangent, and their variation with frequency at room temperature. Figure 11a–c shows the variation of the dielectric permittivity (Figure 11a), conductivity (Figure 11b), and dielectric loss (Figure 11c) of the PU-based composite membranes embedded with different amounts of IONPs. As observed, the permittivity decreases slowly with an increase in the IONPs content. Due to the low amount of IONPs embedded in the PU-based matrix, the PU layer can effectively isolate the IONPs, and the dipole had a tougher time to react to the electric field, resulting in a decrease in permittivity. Moreover, the permittivity values of all the samples suggested a small dependence on the frequency, in the range 10^1–^10^7^ Hz. As a result, the permittivity tends to fall with increasing frequency, showing a tapering off in the number of dipoles that can follow the charge in the electric field.

Conductivity is dependent on frequency, as shown in Figure 11b. Thus, at low frequencies (10^0^–10^3^ Hz), the conductivity of the pure and PU-based composites increases slowly and then increases more sharply up to frequencies of 10^4^ Hz. It was also observed that the conductivity curves in the low frequency range (10^0^–10^3^ Hz) presented a small decrease with the amount of IONPs embedded, while at a high frequency (10^3^–10^7^ Hz) the curves are very close. As previously reported, the small decrease in the conductivity with the increase in the IONPs content is perhaps due to the fact that each IONP can be effectively isolated by the polyurethane layer during preparation, and the conductivity can be suppressed.

The variation of dielectric loss (tan δ) versus frequency in the range from 10^1^ to 10^6^ Hz is shown in Figure 11c. The dielectric loss (tan δ) represents the energy dissipation in the dielectric system, which is considered to be caused by the domain wall resonance [66]. From this figure, it is quite clear that the dielectric loss decreases with the increase in frequency up to 10^4^ Hz and increases slowly up to 10^6^ Hz.

Thus, the quantities of IONPs embedded in the PU matrices tended to diminish the dielectric characteristics, due to various reasons. One of these may be due to the particle size effect, when the small particles of IONPs (in our case <50 nm) lead to a reduction in the electrical dipoles inside the composite membranes, resulting in a decrease in the dielectric properties [13,67]. Another factor is the lowering of the charge carrier mobility with the frequency inside of the membranes, due to coating the IONPs with polyurethane.

The magnetic properties of PU-based composite membranes were also evaluated by using a VSM apparatus. The results of the magnetization measurements as a function of the applied field between −30 and 30 kOe, at room temperature, are reported in Figure 11d. The PU-based membranes without IONPs (Pw) exhibited no magnetic properties due to the absence of magnetic particles in the matrix. When IONPs were added, the magnetization increased with the increase in the IONPs content. Thus, the saturation magnetization values were 0.06, 0.3, and 0.6 emu/g for Pw-0.1, Pw-0.5, and Pw-1, respectively. The values of the saturation magnetization of the magnetic PU-based membranes were low when compared to the neat iron oxide nanoparticles reported in the literature [68]. This is due to the relatively small concentration of IONPs on the one hand and the coating of the IONPs with polyurethane on the other, which results in restricting the rotation of the magnetic moment [14]. Additionally, it should be noted that the maximum magnetization values of the samples were detected at a magnetic field of around 3 kOe. The prepared magnetic PU-based membranes also displayed superparamagnetic properties with low coercivity and remanence (inset of Figure 11d).

Therefore, the magnetic behavior of these PU-based composites has the potential to be exploited in tailoring some electromagnetic devices in biomedical applications, considering that the field required for such applications is 0.2 Tesla (2 kOe) [69]. Furthermore, the low magnetization would not compromise the material characteristics for a tailored application, and the magnetic properties are quite comparable to those of other polyurethane formulations found in the literature [59].

#### 3.2.5. Cytotoxicity Evaluations

**Figure 12:**
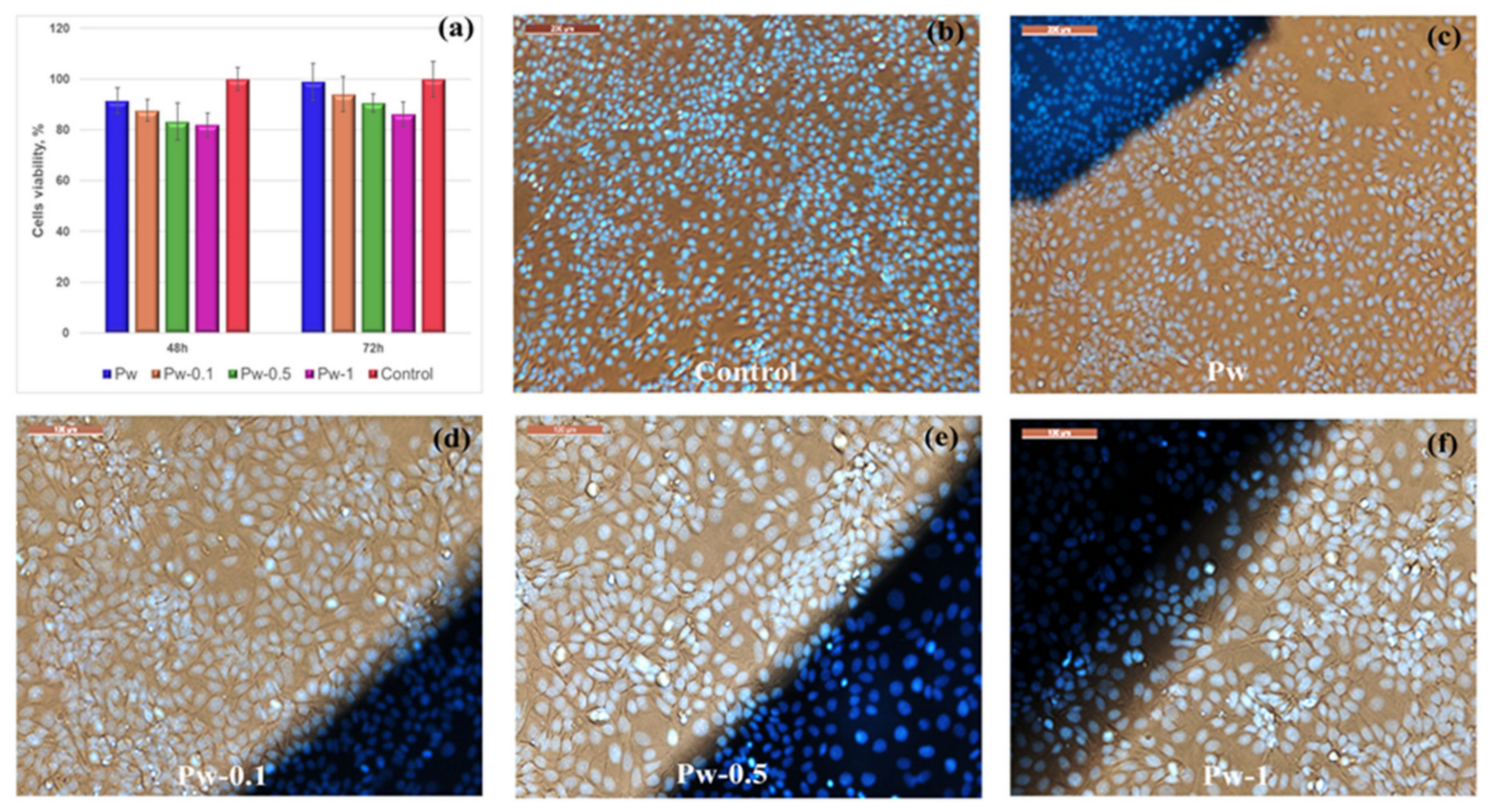
Evaluation of cytotoxicity: (**a**) MTT assay and (**b**–**f**) morphological visualization of epithelial cells after 72 h direct contact with PU-based composite membranes prepared in wet conditions with different amounts of IONPs.

The conventional MTT assay and optical microscope observation were used to evaluate the toxicity of magnetic PU-based composite membranes to epithelial cells (MCF 7-cell-line). The metabolic activity’s dependence on IONPs concentration after 48 and 72 h of incubation in the cell’s medium, as assessed by the MTT assay, is shown in Figure 12a. As observed, the investigated samples presented a cellular viability (%) greater than 80%, so the membranes could be considered non-toxic based on the ISO 10993-5:2009 standard. After 48 h of incubation with cells, the viability of Pw was higher than that of the magnetic composite membranes (Pw-0.1 to Pw-1), though with a decrease in the viability with the increase in the IONPs amount. The IONPs amount influenced the cell viability, such that the lower the IONPs content was, the better the non-cytotoxic properties. This behavior was also observed in our previous studies [13] and in the literature [70]. After 72 h, cell viability increased for all the samples. These results indicate that not only are these membranes non-toxic but also epithelial cells can grow on the membrane surfaces for a long period. This means that the cells can proliferate on the magnetic PU-based composite membranes, and the incorporation of IONPs does not significantly affect the biocompatibility of the studied membranes, even though a small decrease in cell viability was observed with the increasing amount of IONPs.

The morphology and distribution of the cells grown on the prepared PU-based composite membranes after 72 h of incubation were visualized by an optical microscope and compared with the cells on the control plate (Figure 12b–f). The images indicated that the MCF 7 epithelial cells grew in a monolayer and are spread well, with a small density difference between the control and the examined membranes. Additionally, the cells do not change their morphology, retaining the typical shape of the MCF 7 epithelial cell line.

## 4. Conclusions

From the point of view of engineering applications, the properties of the polymer nanocomposites should be controllable or predictable. Therefore, in order to develop membranes with the desired structure, the preparation parameters have to be judiciously chosen, which requires a series of experiments for any new membrane. Thus, in the first part of this study, the optimized protocols in the dry-/wet-phase inversion process have been successfully applied to essentially form defect-free composite membranes, which could be a tremendous contribution to membrane technology. We tried to select the appropriate methods and membrane fabrication systems to produce PU-based composite membranes with the desired morphology and stability and to select the best method to achieve these properties in an efficient way for future research. After an initial study of the structural properties, the best compositions were selected in order to prepare and characterize the final membranes. These conditions included a 20 wt.% PETU-based solution in DMF, the addition of 2 wt.% triblock copolymer Pluronic L-31, and casting by the wet (distilled water bath at 45 °C with a 30 min precoagulation time) solution method.

Then, in the second part, different concentrations of IONPs (0.1, 0.5, and 1 wt.%) were added and the well-prepared magnetic PU-based composite membranes were investigated from the point of view of their morphological, physico-chemical, mechanical, dielectric and magnetic properties. SEM analysis of the surfaces revealed that the membranes prepared under the previously mentioned conditions exhibited a good homogenization of the IONPs and a nice surface porous structure, presenting a uniform distribution of interconnected pores, with diameters between 4 and 20 µm. The profilometric analysis of the surfaces confirms that the roughness of the membrane surface increases by increasing the IONPs content from 605 up to 1411 nm. The addition of IONPs changed the hydrophobicity of the composite membrane surfaces, decreasing it up to 80°. Thus, the hydrophilicity and water vapor permeability of the PU-based composite membranes increases with the IONPs content, due to the establishment of hydrogen and some coordination bonds between the Fe_2_O_3_ complex and -N-H or -C=O groups of the polyurethane structure. The addition of IONPs causes an overall improvement in the tensile properties compared to those of neat polyurethane, achieving a reinforcement effect on the polyurethane matrix. The quantities of IONPs embedded in the PU matrices tended to diminish the dielectric characteristics, due to the particle size effect and coating of the IONPs with polyurethane. These well-prepared magnetic PU-based membranes also displayed superparamagnetic properties with low coercivity and remanence. In addition, their in vitro cytocompatibility was also verified by the MTT assay and their cell morphology. Thus, the investigated samples presented a cell viability greater than 80%, so the membranes could be considered non-toxic. Moreover, after 72 h of incubation, the investigated MCF 7 epithelial cells grew in a monolayer, spread well, did not change their morphology, and kept the typical shape of the cell line.

Since the synthesis and preparation of these PU-based composite membranes were carefully controlled, new external devices can be further prepared according to the envisaged application. Therefore, the results reported here may pave the way for the design of some suitable devices based on these magnetic PU composite membranes with tailored properties, to improve the image quality in magnetic resonance imaging investigations. Thus, by using this device, local image artifacts and blurring could be suppressed.

## Figures and Tables

**Table 1:** WVP coefficients of PU-based magnetic composite membranes.

Sample	G/t (g/h)	R^2^	A (cm^2^)	WVP (g/m^2^h)
Pw	0.0497	0.9981	10	49.7 ± 0.9
Pw-0.1	0.0526	0.9980		52.6 ± 1.2
Pw-0.5	0.0613	0.9979		61.3 ± 1.3
Pw-1	0.0665	0.9973		66.5 ± 1.1

G/t represents the slope of the linear regression of the water loss as a function of time, R^2^ is the correlation coefficient, and A is the area of permeation determined by the mouth cup diameter.

## Data Availability

The data presented in this study are available on request from the corresponding author.
